# Assessment of challenges and opportunities in antibiotic stewardship program implementation in Northwest Ethiopia

**DOI:** 10.1016/j.heliyon.2024.e32663

**Published:** 2024-06-07

**Authors:** Asrat Agalu Abejew, Gizachew Yismaw Wubetu, Teferi Gedif Fenta

**Affiliations:** a Department of Pharmaceutics and Social Pharmacy, School of Pharmacy, College of Health Sciences, Addis Ababa University, Addis Ababa, Ethiopia; b Department of Pharmacy, College of Medicine and Health Sciences, Bahir Dar University, Bahir Dar, Ethiopia; c Amhara Public Health Institute, Bahir Dar, Ethiopia

**Keywords:** Antibiotic resistance, Antibiotic stewardship, Belief, Antibiotic prescribing, Readiness

## Abstract

**Background:**

Indiscriminate use of antibiotics leads to antibiotic resistance (AMR) and results in mortality, morbidity, and financial burden. Antibiotic stewardship programs (ASPs) with education can resolve a number of barriers recognized in the implementation of successful ASPs. The aim of this study was to assess health professionals’ perceptions and status of ASPs in hospitals in 2022.

**Methods:**

A cross-sectional study was conducted from September 1, 2022 to October 30, 2022. A total of 181 health professionals were included, and a self-administered questionnaire was used to collect data. The status of hospitals was assessed using a checklist. The data were analyzed using SPSS version 23, and descriptive statistics and Chi-square tests (X^2^) at a P-value of <0.05 were used.

**Results:**

Of the 181 respondents, 163 (90.1 %), and 161 (89.0 %) believed that AMR is a significant problem in Ethiopia and globally, respectively. Easy access to antibiotics 155 (85.6 %), and inappropriate use 137 (75.7 %) were perceived as key contributors to AMR. Antibiotics were believed to be prescribed/dispensed without laboratory results 86 (47.5 %), and antibiotic susceptibility patterns were not considered to guide empiric therapy 81 (44.8 %). ASP was believed to reduce the duration of hospital stays and associated costs 137 (75.7 %), and improve the quality of patient care 133 (73.5 %), whereas 151 (83.4 %), 143 (79 %), and 142 (78.5 %) suggested education, institutional guidelines, and prospective audits with feedback interventions to combat AMR in their hospitals, respectively. There were significant differences in perception among professionals based on professional category and attempts by hospitals to implement ASPs. Although ASPs were not functioning according to standard, there have been attempts to implement it in three hospitals. The issue of ASP had never been heard in general hospitals. Currently, it is feasible to implement ASPs in four hospitals.

**Conclusion:**

The status of ASP in hospitals was very poor. Despite a lack of prior knowledge on ASPs, most respondents do have a positive perception of AMR and the implementation of ASPs. Pharmacist-led prospective audits and feedback with education and institutional guidelines for empiric antibiotic use can be better implemented in hospitals. Involvement of representatives from infection prevention and control, and collaboration among hospitals in ASP implementation will help establish a strong ASP in the area.

## 1 Introduction

Antibiotics have saved millions of lives and transformed modern medicine [1,2]. Although the leading cause of death from infectious diseases was drastically reduced [[1], [2], [3], [4]], about 30–50 % of antibiotic prescriptions in hospitals were used either unnecessarily or inappropriately. It was indicated that global antibiotic consumption was drastically increasing from time to time due to increased consumption of antibiotics in low- and middle-income countries (LMICs) [5]. The increased consumption of antibiotics toppled by inappropriate consumption has resulted in the ineffectiveness of antibiotics due to the development of AMR [2,[6], [7], [8], [9], [10], [11], [12], [13], [14], [15]]. These could have resulted in treatment failure and an increased risk of adverse patient outcomes, such as *Clostridium* difficile infections and other adverse events [1,4,16]. AMR is a global public health treat resulting in morbidity and mortality [1,2,[17], [18], [19]]. The LMICs are disproportionately affected by AMR, which is a serious problem for the poorest countries due to different reasons [17,[20], [21], [22], [23], [24], [25]]. Despite the high level of AMR and attempts to implement ASPs, in LMICs, evidence on effective and feasible stewardship interventions is still limited, and challenges for the implementation of interventions are numerous [[26], [27], [28], [29]].

These call for locally contextualized strategies while identifying targeted and sustainable indicators to track antibiotic use, AMR, infection prevention and control, and improving access to antibiotics and reducing inappropriate use [[30], [31], [32], [33], [34]]. In hospital settings, inappropriate antibiotic prescribing is a key contributor to the emergence of AMR, which itself is often driven by patient demand for antibiotics, misaligned economic incentives, a lack of knowledge of appropriate antibiotic prescribing, and/or a lack of and delayed laboratory results to assist antibiotic prescribing [35,36], affecting the antibiotic prescribing behaviors of physicians [37,38]. Prescribing behavior is affected by social and cultural norms and attitudes toward prescribing, resulting in irrational prescribing of antibiotics and increased consumption of reserve antibiotics, such as carbapenems [5,39,40]. High antibiotic prescriptions may be related to poor knowledge, a lack of awareness of AMR, and limited motivation to change [38,41].

In most sub-Saharan African countries, AMR has not been a priority, even for those whose customized national AMR action plans (NAP) are developed based on global AMR action plans (GAP), the implementation is still inadequate [29,30,42], despite the high burden of AMR [17,31]. Although ASPs are recognized as formal strategies for curbing increased trends in AMR [43], ASP preparedness in Sub-Saharan Africa was reported to be very poor and varies greatly from country to country, ranging from the inability to determine the building blocks to support ASPs to those that have systems that allow ASP integration into clinical practices [28]. ASP strategies are not uniformly implemented across health facilities in those countries that have implemented ASP [44,45], indicating that the LMICs, including Africa, are facing multiple challenges and barriers to implementing ASP [[43], [44], [45], [46], [47], [48], [49], [50]]. As a requirement for ASP implementation, determining the challenges and understanding the situation of health facilities is essential [4,43,44,51]. Since physicians and pharmacists are essential components of ASP teams for the successful implementation of ASPs, examining their perspectives about the dynamics within their team and contextual factors that facilitate the success programs is also a component and first step for ASP implementation [51]. This helps create awareness about AMR, develop and maintain guidelines for antibiotic use, monitor antibiotic use, ensuring the quality of low-cost generic medicines, and improve laboratory services for the successful implementation of ASP in hospitals [33].

In Ethiopia, AMR was taken as a priority [52], although ASP was not officially implemented yet [53], despite preparing ASP implementation guidelines [3]. Currently, there are only individual attempts to implement ASP [10,44] and pilot quality improvement interventions to optimize the use of antimicrobials for surgical prophylaxis in some hospitals [54]. Thus, this study is aimed at determining the gaps and readiness of hospitals for ASP implementation and the perception of health professionals about antibiotic resistance and ASPs in the inpatient wards of hospitals in Northwest Ethiopia.

## 2 Methods and materials

### 2.1 Study design, study setting and period

A cross-sectional study was conducted from September 2022 to October 2022 in five hospitals. The hospitals are Felege Hiwot Comprehensive Specialized Hospital (FHCSH), Tibebe Ghion Specialized Hospital (TGSH), Debre Markos Comprehensive Specialized Hospital (DMCSH), Finote Selam General Hospital (FSGH), and Injibara General Hospital (IGH). These hospitals are among the 81 hospitals in the Amhara National Regional State. The clinical service for infectious diseases in all health facilities in the region is mostly carried out based on clinical diagnosis and complete blood count (CBC) results. There are no rapid diagnostic techniques for the identification of bacterial infections in hospitals. The hospitals provide integrated services to patients in their outpatient departments, emergency triage assessment and treatment departments, inpatient departments, and neonatal intensive care units. Except for the attempts to assign patient-oriented pharmacists in some hospitals, there are no ASPs and clinical pharmacy services integrated into the hospital's patient care systems. This readiness assessment of hospitals to determine gaps for implementation of ASPs and a survey of health professionals to determine perceptions on AMR, antibiotic prescribing and use, ASPs, and possible solutions was part of broader data collection for gap analysis and baseline data collection, including trends in infectious diseases and antibiotic consumption and use in the pre-implementation phase of ASPs.

### 2.2 Study participants

About 200 health professionals were included in the study. Physicians working in the medical ward, pediatric ward, gynecology/obstetrics ward, and surgical ward were included in the study. In addition, pharmacy professionals who engaged in the dispensing of antibiotics in hospitals were also included in the study. Data on the readiness of hospitals was collected from those who do have full information about the situation of hospitals, such as medical directors, human resource personnel, laboratory personnel, and leaders of ASPs, using checklists.

### 2.3 Sample size and sampling procedures

The sample size for health professionals was determined based on the following formula for finite population:

n = *Χ*^2^*NP* (1-*P*)/ (*d*^2^ (*N* – 1) + *Χ*^2^*P* (1 - *P*))

Where n is the sample size and X^2^ is the table value of the chi-square for 1 degree of freedom at the desired confidence level (1.96*1.96 = 3.841); N is the total population; P is the population proportion (27 %) and d is the degree of accuracy expressed as a proportion (0.05). Gebretekle et al. [44] reported a prevalence that ranges from 27 % for perceived antibiotic prescription or dispensing to 90 % for AMR. Thus, a prevalence of 27 % was used to provide the maximum sample size in this study.

Accordingly, n, *Χ*^2^N*P* (1 - *P*)/(*d*^2^ (*N* – 1) + *Χ*^2^*P* (1 - *P*)) was calculated as follows:

= (1.96*1.96) *487*0.27(1–0.27)/ ((0.05*0.05) *(487–1) + (1.96*1.96) *0.27(1–0.27))

=181.26–∼182

To account for the nonresponse rate, 10 % was added, and thus, 200 health professionals were included in the study and allocated to hospitals proportionally based on the estimated number of physicians and pharmacy professionals in each hospital.

### 2.4 Data collection instruments and processes

A data collection tool used by Gebretekle et al. [44] to explore healthcare providers’ knowledge and perceptions on AMR, barriers, and facilitators to successful implementation of a pharmacist-led ASP intervention in a referral hospital in Ethiopia was used. In addition, the capacity and readiness of health facilities were assessed based on the core elements of hospital ASPs [55]. The questionnaire was designed on a Likert scale with a 6-point response format, which ranges from 1 (strongly disagree) to 5 (strongly agree) and 6 (not applicable). The questionnaire consists of experiences with antibiotic use, perceptions about the scope of antibiotic resistance, the causes of antibiotic resistance, and the possible barriers to compliance with infection control precautions; strategies for antibiotic resistance prevention; and sources for information about antibiotic use. All physicians from the inpatient wards of internal medicine, surgery, pediatrics and gynecology/obstetrics, and pharmacy professionals working in the hospital were approached to participate in the study. The questionnaire was distributed in morning sessions, grand rounds, and/or while at work for physicians, and pharmacists were approached at their worksite during working hours. The completeness of the data was monitored on a daily basis.

### 2.5 Data analysis

Before the analysis of the quantitative data, the outcome metrics were first determined, and the data was coded, entered into SPSS version 21, and finally cleared and analyzed. Descriptive statistics were used to characterize the variables of interest. The degree of agreement with the statements was reclassified into “agree” (strongly agree/agree), “disagree” (strongly disagree/disagree), and “neutral” (neutral/NA). The χ^2^ tests with a significance level of p < 0.05 were employed to explore differences in proportions of agreement between physicians and pharmacists and among health professionals from hospitals that attempted to implement ASPs and those that didn't. The results were finally presented in tables based on the nature of the results.

### 2.6 Ethical consideration

Ethical approval was obtained from Addis Ababa University, the College of Health Sciences ((protocol code: 106/22/SoP), and the School of Pharmacy (protocol code: ERB/SOP/472/14/2022). A support letter to the hospitals was obtained from the Amhara Public Health Institute. All participants provided informed consent prior to participating in this study. The information obtained was kept confidential and used only for research purposes. Ethical issues were considered during data collection in order not to disclose information to people outside the research.

## 3 Results

Five hospitals were included in the assessment of the current situation, and a total of 181 (90.5 %) of the respondents returned the questionnaire among the 200 distributed. Those who didn't complete the questionnaires on time and returned them were considered non-responders. The results for the perception of health professionals on AMR, antibiotic use, and ASP, as well as the current situation and status of ASP in hospitals, including the core elements of ASP, were described.

A. Perceptions of health professionals on AMR, antibiotic use, and ASP

### 3.1 Socio-demographic characteristics of respondents

The majority of health professional participants, 122 (75.8 %), were greater than 29 years old with a mean age of 32 ± 4.3 (range 23–45 years old), and 124 (77 %) were male. The respondents reported that they had treated or dispensed on average to 254 patients per week, among whom 69.7 % believed to prescribe or dispense antibiotics, during a one-week period (Table 1).

**Table 1:** Socio-demographic characteristics of health professionals in the Hospitals, in 2022, (N = 181).

Variables		Frequency (%)
Age in years	18–29	46 (25.4)
	30–64	135 (74.6)
	Total	181 (100)
Sex	Male	138 (76.2)
	Female	43 (23.8)
	Total	181 (100)
Facility	FHRH	46 (25.4)
	TGSH	68 (37.6)
	DMCSH	31 (17.1)
	IGH	16 (8.8)
	FSGH	20 (11)
	Total	181 (100)
Work area	Medicine	29 (16)
	Surgery	29 (16)
	Pediatrics	28 (15.5)
	Gyne/obs	20 (11)
	Pharmacy	75 (41.4)
	Total	181 (100)
Position/profession	Physician	106 (58.6)
	Pharmacy	75 (41.4)
	Total	181 (100)
Work experience in the current hospital	Overall	3.7 ± 2.3
	<5 years	137 (75.7)
	≥5 years	44 (24.3)
	Total	181 (100)
Work experience in the current position	Overall	4.5 ± 4.1
	<5 years	123 (68)
	≥5 years	58 (32)
	Total	181 (100)
Average number patients treated/dispensed per week	34997	254 ± 198
Average number patients treated or dispensed with at least one antibiotic per week among visits	23415 (66.9 %)	204 ± 135

### 3.2 Believe of health professionals on the scope of AMR

From 181 health professionals, 163 (90.1 %), and 161 (89.0 %) agreed on the statements that AMR is a significant problem in Ethiopia and worldwide, respectively. But only 53 (29.3 %), and 52 (28.7 %) agreed with statements that a very high proportion (>30 %) of MRSA and a very high proportion (>30 %) of gram-negative infections are highly drug-resistant in this hospital, respectively. Pharmacy professionals believe more in a statement that a high proportion of gram-negative infections are highly drug-resistant to all cephalosporins, and some are even resistant to carbapenems (P-value = 0.035), and a very high proportion of staphylococcal infections are resistant to methicillin (MRSA) in their hospitals (P-value = 0.0001) (Table 2). The overall response of health professionals’ beliefs on AMR, antibiotic use, ASP, and potential solutions is presented in Supplementary Table 1.

**Table 2:** Perception of health professionals on scope of antibiotic resistance in the hospitals, in 2022, (N = 181).

Items and Level of agreement on each statement		Category of Profession			P-value
		Physician (%)	Pharmacy (%)	Total (%)	
Antibiotic resistance is a significant problem worldwide	Disagree	7(0.5)	7 (0.5)	14 (7.7)	0.712
	Neutral	3(0.5)	3(0.5)	6 (3.3)	
	Agree	96 (59.6)	65(40.4)	161 (89.0)	
Antibiotic resistance is a significant problem in my country	Disagree	5 (45.5)	6(54.5)	11 (6.1)	0.532
	Neutral	5(71.4)	2(28.6)	7 (3.9)	
	Agree	96(58.9)	67(41.1)	163 (90.1)	
Antibiotic resistance is a significant problem in my hospital	Disagree	4 (40)	6(60)	10 (5.5)	0.23
	Neutral	29(67.4)	14(32.6)	43 (23.8)	
	Agree	73(57.0)	55(43.0)	128 (70.7)	
Antibiotic resistance is a problem in my daily practice	Disagree	10(52.6)	9(47.3)	19 (10.5)	0.84
	Neutral	32(60.4)	21(39.6)	53 (29.3)	
	Agree	64(58.7)	45(41.2)	109 (60.2)	
A patient is likely to develop drug-resistant infection during their hospital stay at this hospital	Disagree	19(63.3)	11(36.7)	30 (16.6)	0.842
	Neutral	25(58.1)	18(41.9)	43 (23.8)	
	Agree	62(57.4)	46(42.6)	108 (59.7)	
I think a very high proportion (>30 %) of gram-negative infections are highly drug- resistant in this hospital (resistant to all cephalosporins, and some are even resistant to carbapenems)	Disagree	19(70.3)	8(29.7)	27 (14.9)	0.035^a^
	Neutral	64(62.7)	38(36.3)	102 (56.4)	
	Agree	23(44.2)	29(55.8)	52 (28.7)	
I think a very high proportion (>30 %) of Staphylococcal infections are resistant b 0020to methicillin (MRSA) in this hospital	Disagree	24(72.7)	9(27.3)	33 (18.2)	0.001^a^
	Neutral	62(65.3)	33(34.7)	95 (52.5)	
	Agree	20(37.7)	33(62.3)	53 (29.3)	

*Physicians more agreed.

a Pharmacy professionals agreed.

### 3.3 Beliefs of health professional on factors contributing to AMR

The majority, 155 (85.6 %) and 137 (75.7 %) of professionals, agreed that easy access to antibiotics and inappropriate use were key contributors to AMR, respectively. But only 19 (10.5 %) and 22 (12.2 %) of health professionals perceive that the staff education on antibiotic consumption and drug-resistant organisms and the performance of adequate surveillance for drug-resistant organisms are adequate, respectively. There was a significant difference between physicians and pharmacists on the prescription of broad-spectrum antibiotics, which is directly linked to AMR (P-value = 0.045), patient demands and expectations contribute to the overuse of antibiotics (P-value = 0.003), and poor infection control practices by health professionals significantly contribute to the spread of AMR (P-value = 0.022), all of which were more prevalent in pharmacists (Table 3).

**Table 3:** Beliefs on possible factors contributing to antibiotic resistance in the hospitals, in 2022 (N = 181).

Items and Level of agreement on each statement, frequency (%)		Category of Profession			P-value
		Physician (%)	Pharmacy (%)	Total (%)	
Inappropriate consumption of antibiotics is a major cause of antibiotic resistance in this hospital	Disagree	9(64.3)	5(35.7)	14 (7.7)	0.31
	Neutral	21(70.0)	9(30.0)	30 (16.6)	
	Agree	76(55.5)	61(44.5)	137 (75.7)	
The easy access to antibiotics without a prescription in Ethiopia contributes to antibiotic resistance	Disagree	8(53.3)	7(42.7)	15 (8.3)	0.259
	Neutral	9(81.8)	2(19.2)	11 (6.1)	
	Agree	89(57.4)	66(42.6)	155 (85.6)	
The prescription of broad-spectrum antibiotics is directly linked to antibiotic resistance in this hospital	Disagree	20(69.0)	9(31.0)	29 (16)	0.045^a^
	Neutral	34(70.8)	14(29.2)	48 (26.6)	
	Agree	52(50.0)	52(50.0)	104 (57.5)	
This hospital performs adequate surveillance for drug-resistant organisms	Disagree	80(63.0)	47(37.0)	127 (70.2)	0.144
	Neutral	18(51.4)	17(47.6)	35 (19.3)	
	Agree	8(42.1)	11(57.9)	19 (10.5)	
The lack of adequate diagnostic tests in this hospital leads to overuse of antibiotics contributing to AMR	Disagree	22(68.8)	10(31.2)	32 (17.7)	0.117
	Neutral	13(43.3)	17(56.7)	30 (16.6	
	Agree	71(59.7)	48(40.3)	119 (65.7	
This hospital provides adequate staff education regarding antibiotic consumption and resistance	Disagree	79(63.7)	45(36.3)	124 (68.5)	0.048^a^
	Neutral	19(54.3)	16(45.7)	35 (19.3)	
	Agree	8(36.4)	14(63.6)	22 (12.2)	
I suspect that antibiotics available in my hospital are of poor quality and contribute to AMR	Disagree	56(55.4)	45(44.6)	101(55.8)	0.547
	Neutral	32(60.4)	21(39.6)	53 (29.3)	
	Agree	18(66.7)	9(33.3)	27 (14.9)	
	Neutral	18(52.9)	16(47.1)	34 (18.8)	
	Agree	69(62.2)	42(37.8)	111 (61.3)	
The sporadic supply of antibiotics in my hospital leads to interruptions of therapy contributing to antibiotic resistance	Disagree	24(60.0)	16(40.0)	40 (22.1)	0.534
	Neutral	26(52.0)	24(48.0)	50 (27.6)	
	Agree	56(61.5)	35(38.5)	91 (50.3)	
The lack of close clinical follow-up during antibiotic consumption in my hospital contributes to antibiotic resistance	Disagree	25(73.5)	9(26.5)	34 (18.8)	0.182
	Neutral	24(58.5)	17(41.5)	41 (22.6)	
	Agree	57(53.8)	49(46.2)	106 (58.6)	
Patient demands and expectations contribute to overuse of antibiotics contributing to AMR in this hospital	Disagree	36(67.9)	17(32.1)	53 (29.3)	0.003^a^
	Neutral	42(70.0)	18(30.0)	60 (33.2)	
	Agree	28(41.2)	40(58.8)	68 (37.6)	
	Neutral	14(53.8)	12(46.2)	26 (14.4)	
	Agree	81(64.3)	45(35.7)	126 (69.6)	
Poor infection control practices by health professionals significantly contributes to the spread of antibiotic resistance in this hospital	Disagree	19(52.8)	17(47.2)	36 (19.9)	0.022^a^
	Neutral	31(77.5)	9(22.5)	40 (22.1)	
	Agree	56(53.3)	49(46.7)	105 (58.)	
Adherence to hand-hygiene protocols is acceptable at this hospital	Disagree	48(69.6)	21(29.4)	69 (38.1)	0.044^a^
	Neutral	27(56.3)	21(43.7)	48 (26.5)	
	Agree	31(48.4)	33(51.6)	64 (35.4)	
Patient rooms and equipment are cleaned appropriately as per hospital cleaning protocol once a patient carrying a drug-resistant organism (DRO) has been discharged from this hospital	Disagree	60(66.7)	30(33.3)	90 (49.7)	0.007^a^
	Neutral	32(60.4)	21(39.6)	53 (29.3)	
	Agree	14(36.8)	24(64.2)	38 (21.)	

*Physicians more agreed.

a Pharmacy professionals agreed.

### 3.4 Belief of health professionals on antibiotics use

The majority of health professionals 104 (57.5 %) and 101 (55.8 %) had agreed only on two statements: I routinely try to step down intravenous antibiotics to an oral alternative antibiotic after about three days, and I routinely prescribe or dispensing very broad-spectrum antibiotics empirically because microbiology lab results are not available in a timely fashion, respectively. But 86 (47.5 %), 84 (46.4 %), and 81 (44.8 %) disagreed about routinely checking microbiology laboratory results to guide antibiotic choice, timely communication of microbiology lab results, and using evidence-based empiric antibiotic prescribing practices, respectively. There were differences between physicians and pharmacists on cost considerations (P-value = 0.03) and routinely stepping down intravenous antibiotics to an oral alternative antibiotic (P-value = 0.021), which was more favored by physicians, but recommending very broad-spectrum antibiotics empirically because microbiology lab results are not available in a timely fashion (P-value = 0.04) by pharmacy professionals (Table 4).

**Table 4:** Belief of health professionals on antibiotics prescription/dispensing practices in the hospitals, in 2022 (N = 181).

Items and Level of agreement on each statement, frequency (%)		Category of profession			P-value
		Physician (%)	Pharmacy (%)	Total (%)	
Microbiology lab results are timely communicated to the health professionals in this hospital	Disagree	53(63.1)	31(36.9)	84 (46.4)	0.043^b^
	Neutral	36(64.3)	20(35.7)	56 (30.9)	
	Agree	17(41.5)	24(58.5)	41 (22.7)	
My choice of antibiotics is often influenced by the availability of the antibiotics rather than by the local antibiogram or by the etiologic cause of disease (availability of laboratory results)	Disagree	19(52.8)	17(47.2)	36 (19.9)	0.465
	Neutral	18(52.9)	16(47.1)	34 (18.8)	
	Agree	69(62.2)	42(37.8)	111 (61.3)	
Cost considerations for the patient affects my choice of antibiotics	Disagree	11(37.9)	18(62.1)	29 (16)	0.03^a^
	Neutral	14(53.8)	12(46.2)	26 (14.4)	
	Agree	81(64.3)	45(35.7)	126 (69.6)	
I regularly refer to/consider the antibiotic susceptibility patterns at this hospital (institutional antibiogram) when empirically prescribing or recommending antibiotics	Disagree	51(63.0)	30(37.0)	81 (44.8)	0.007^b^
	Neutral	38(67.9)	18(32.1)	56 (30.9)	
	Agree	17(38.6)	27(61.4)	44 (24.3)	
If medically appropriate, I routinely try to step down intravenous antibiotics to an oral alternative antibiotic after about three days	Disagree	16(41.0)	23(59.0)	39 (21.5)	0.021^a^
	Neutral	21(55.3)	17(44.7)	38 (21)	
	Agree	69(66.3)	35(33.7)	104 (57.5)	
If medically appropriate, I routinely try to step down broad-spectrum antibiotics to a narrow-spectrum antibiotic after about three days	Disagree	36(58.1)	26(41.9)	62 (34.3)	0.995
	Neutral	30(58.8)	21(41.2)	51 (28.2)	
	Agree	40(58.8)	28(41.2)	68 (37.6)	
Restrictions on antibiotics could impair my ability to provide good patient care	Disagree	43(67.2)	21(32.8)	64 (35.4)	0.08
	Neutral	24(63.2)	14(36.8)	38 (21)	
	Agree	39(49.4)	40(50.6)	79 (43.6)	
I routinely prescribe/recommend very broad-spectrum antibiotics empirically because I believe most patients are infected with a drug-resistant organism	Disagree	55(71.4)	22(28.6)	77 (42.5)	0.004^b^
	Neutral	27(56.3)	21(43.7)	48 (26.5)	
	Agree	24(42.9)	32(57.1)	56 (30.9)	
I routinely prescribe/recommend very broad-spectrum antibiotics empirically because microbiology lab results are not available in a timely fashion	Disagree	40(71.4)	16(28.6)	56 (30.9)	0.029^b^
	Neutral	10(41.7)	14(58.3)	24 (13.3)	
	Agree	56(55.4)	45(44.6)	101 (55.8)	
I routinely check microbiology laboratory results to guide my choice of antibiotics	Disagree	56(62.9)	30(37.1)	86 (47.5)	0.155
	Neutral	26(57.8)	19(42.2)	45 (24.9)	
	Agree	24(48.0)	26(52.0)	50 (27.6)	
In the past seven days, I have prescribed broad spectrum antibiotics for longer than 3 days for fewer than 10 % of my patients;	Disagree	42(60.0)	28(40.0)	70 (38.7)	0.013^a^
	Neutral	27(45.0)	33(55.0)	60 (33.1)	
	Agree	37(72.5)	14(27.5)	51 (28.2)	
In the past seven days, I have prescribed broad spectrum antibiotics for longer than 3 days for 10–50 % of my patients	Disagree	46(66.7)	23(33.3)	69 (38.1)	0.201
	Neutral	31(51.7)	29(48.3)	60 (33.1)	
	Agree	29(55.8)	23(44.2)	52 (28.7)	
In the past seven days, I have prescribed/recommended broad spectrum antibiotics for longer than 3 days for more than 50 % of my patients	Disagree	54(74.0)	19(26.0)	73 (40.3)	0.003^b^
	Neutral	27(48.2)	29(51.8)	56 (30.9)	
	Agree	25(48.1)	27(51.9)	52 (28.7)	

a Physicians more agreed.

b Pharmacy professionals agreed.

### 3.5 Perception of health professionals on implementation of ASP

The majority of health professionals, 151 (83.4 %), 137 (75.7 %), and 133 (73.5 %) believe that education on antibiotic therapy for physicians and pharmacy staffs, ASP reduces the duration of hospital stays and associated costs, and ASP improves the quality of patient care, respectively. Only 29 (16.0 %), and 39 (2.2 %) of professionals agreed that ASPs can be an obstacle to good patient care, and prescribing physicians are the only professionals who need to understand ASPs, respectively. Pharmacists believe that investing more in ASP reduces overall antibiotic consumption and can result in cost savings (P-value = 0.049), but they insist that they do not have enough time to invest in ASP (P-value = 0.03) (Table 5).

**Table 5:** Perception of health professionals on implementation of ASP in the hospitals, in 2022 (N = 181).

Items and Level of agreement on each statement		Category of Profession			P-value
		Physician (%)	Pharmacy (%)	Total (%)	
ASPs (ASP) improve quality of patient care	Disagree	5(55.6)	4(44.4)	9 (5)	0.929
	Neutral	22(56.4)	17(43.6)	39 (21.5)	
	Agree	79(59.4)	54(41.6)	133 (73.5)	
ASP reduces overall antibiotic consumption and can result in cost savings	Disagree	5(33.3)	10(66.7)	15 (8.3)	0.049^a^
	Neutral	26 (70.3)	11(29.7)	37 (20.4)	
	Agree	75(58.1)	54(41.9)	129 (71.3)	
ASP reduces duration of hospital stay and associated costs	Disagree	11(55.0)	9(45.0)	20 (11)	0.94
	Neutral	14(58.3)	10(42.7)	24 (13.3)	
	Agree	81(59.1)	56(41.9)	137 (75.7)	
ASP reduce the problem of antibiotic resistance	Disagree	11(55.0)	9(45.0)	20 (11)	0.934
	Neutral	21(60.0)	14(40.0)	35 (19.3)	
	Agree	74(58.7)	52(41.3)	126 (69.6)	
ASP has impact on an institution's nosocomial infection rates	Disagree	9 (60.0)	6 (40.0)	15 (8.3)	0.369
	Neutral	24 (50.0)	24 (50.0)	48 (26.5)	
	Agree	73 (61.9)	45 (39.1)	118 (65.2)	
This hospital has the capacity to establish and implement an effective ASP	Disagree	23 (65.7)	12 (34.3)	35 (19.3)	0.149
	Neutral	35 (66.0)	18 (34.0)	53 (29.3)	
	Agree	48 (51.6)	45(48.4)	93 (51.4)	
My individual efforts in antibiotic stewardship can significantly impact this hospital's antibiotic resistance problem	Disagree	15 (57.7)	11(42.3)	26 (14.4)	0.844
	Neutral	31 (62.0)	19 (38.0)	50 (27.6)	
	Agree	60(57.1)	45(42.9)	105 (58.0)	
I would like to receive more feedback on my antibiotic selections	Disagree	12(50)	12(50)	24 (13.3)	0.141
	Neutral	17(41.2)	19(58.6)	36 (19.9)	
	Agree	77(63.6)	44(36.4)	121 (66.9)	
ASPs can be an obstacle to good patient care	Disagree	66(59.5)	45(40.5)	111 (61.3)	0.428
	Neutral	26(63.4)	15(36.6)	41 (22.7)	
	Agree	14(48.3)	15(51.7)	29 (16)	
ASPs override prescribers' decision autonomy	Disagree	50(60.2)	33(39.8)	83 (45.9)	0.086
	Neutral	40(65.6)	21(34.4)	61 (33.7)	
	Agree	16(43.2)	21(56.8)	37 (20.4)	
I do not have enough time to further invest into ASP	Disagree	53(55.8)	42(44.2)	95 (52.5)	0.03^a^
	Neutral	40(71.4)	16(28.6)	56 (30.9)	
	Agree	13(43.3)	17(56.7)	30 (16.6)	
Infectious diseases experts that can provide guidance in antibiotic selection and prescription are available in this hospital	Disagree	59(65.6)	31(34.4)	90 (49.7)	0.002^a^
	Neutral	29(67.4)	14(33.6)	43 (23.8)	
	Agree	18(37.5)	30(62.5)	48 (26.5)	
Additional staff education on antibiotic prescribing and use is needed in this hospital	Disagree	13(41.9)	18(58.1)	31 (17.1)	0.118
	Neutral	17(63.0)	10(37)	27 (14.9)	
	Agree	76(61.8)	47(38.2)	123 (67.9)	
Prescribing physicians are the only professionals who need to understand antibiotic stewardship	Disagree	78 (61.9)	48(38.1)	126 (69.6)	0.384
	Neutral	8(50.0)	8(50.0)	16 (8.9)	
	Agree	20(51.3)	19(48.7)	39 (2.2)	
Pharmacists with sufficient training to provide guidance on antibiotics (ex. Antibiotic switches, IV to PO step-down, renal dose adjustments) are available in this hospital	Disagree	52(60.5)	34(39.5)	86 (47.5)	0.003^a^
	Neutral	32(76.2)	10(23.8)	42 (23.2)	
	Agree	22(41.5)	31(58.5)	53 (29.3)	
Implementation of electronic medical recording (e.g., receiving results electronically) can improve effect of ASP	Disagree	9(64.3)	5(35.7)	14 (7.7)	0.757
	Neutral	29(61.7)	18(38.3)	47 (26.0)	
	Agree	68(56.7)	52(43.3)	120 (66.3)	

*Physicians more agreed.

a Pharmacy professionals agreed.

### 3.6 Belief on potential interventions to combat AMR

Among 181 professionals, 151 (83.4 %), 143 (79 %), 142 (78.5 %), and 137 (75.7 %) believe that education on antibiotic therapy to medical and pharmacy staff, developing new institutional guidelines for empiric antibiotic use, implementation of prospective audit and feedback, and active involvement of the hospital infection prevention and control (IPC) team are effective potential interventions to combat AMR (Table 6).

**Table 6:** Belief of health professionals on potential intervention to combat AMR in the hospitals, 2022 (N = 181).

Item and level of agreement		Category of Profession			P-value
		Physician (%)	Pharmacy (%)	Total (%)	
Education on antibiotic therapy to medical and pharmacy staff	Not useful	5 (41.7 %	7 (58.3)	12 (6.6)	0.466
	Unsure	11(61.1)	7(29.9)	18 (9.9)	
	Effective	90(59.6)	61(39.4)	151 (83.4)	
Develop new institutional guidelines for empiric antibiotic use	Not useful	6(37.5)	10(63.5)	16 (8.8)	0.192
	Unsure	14(63.6)	8(36.4)	22 (12.2)	
	Effective	86(60.1)	57(39.9)	143 (79.0)	
Access to institution-specific antibiogram to treating teams	Not useful	3(21.4)	11(79.6)	14 (7.7)	0.013^a^
	Unsure	18(60.0)	12(40.0)	30 (16.6)	
	Effective	85(62.0)	52(38.0)	137 (75.5)	
Implementation of prospective audit and feedback (multidisciplinary rounds on appropriate prescribing and use of antibiotics)	Not useful	4(40.0)	6(60)	10 (5.5)	0.468
	Unsure	17(58.7)	12(41.3)	29 (16)	
	Effective	85(59.9)	57(60.1)	142 (78.5)	
Active involvement of hospital infection prevention and control team	Not useful	5(38.5)	8(61.5)	13 (7.2)	0.301
	Unsure	18(58.1)	13(41.9)	31 (17.1)	
	Effective	83(60.1)	54(39.9)	137 (75.7)	
Antibiotic cycling intervention (e.g., scheduled rotation of 3rd or 4th generation cephalosporins with carbapenems and piperacillin-tazobactam for pre-determined time periods)	Not useful	8(80.0)	2(20.0)	10 (5.5)	0.047^b^
	Unsure	43(67.2)	21(32.8)	64 (35.4)	
	Effective	55(51.4)	52(49.6)	107 (59.2)	
Antibiotic restriction intervention (certain antibiotics cannot be prescribed without infectious disease specialist approval for restricted antibiotics)	Not useful	16(51.6)	15(48.4)	31 (17.1)	0.143
	Unsure	30(71.4)	12(28.6)	42 (23.2)	
	Effective	60(55.6)	48(44.4)	108 (59.7)	

a Physicians deemed as effective strategy.

b Pharmacy professionals deemed as effective.

### 3.7 Difference in level of agreement of health professionals in hospitals attempted ASP

There is difference in the level of agreement among professionals working in hospitals who attempted to implement ASP and those which didn't on the items antibiotic resistance is a significant problem worldwide (P-value = 0.013), inappropriate consumption of antibiotics is a major cause of antibiotic resistance in this hospital (P-value = 0.034) and the easy access to antibiotics without a prescription in Ethiopia contributes to antibiotic resistance (P-value = 0.003) all of which were better agreed by professionals in hospitals which attempted ASP implementation. The lack of adequate diagnostic tests in this hospital leads to overuse of antibiotics (P-value = 0.035) and my choice of antibiotics is often influenced by the availability of the antibiotics (P-value = 0.012) were better agreed by health professionals in hospitals which didn't attempted ASP implementation (Table 7).

B. Situational analysis of hospitals for implementation of ASP

**Table 7:** Difference in level of agreement by attempts to implement ASP in the hospitals, in 2022 (N = 181).

Items and level of agreement for each items		Hospitals attempted ASP			P-value
		No (%)	Yes (%)	Total (%)	
Antibiotic resistance is a significant problem worldwide	Disagree	7(50.0)	7(50.0)	14(7.7)	0.013^a^
	Neutral	1(16.7))	5(83.3)	6(3.3)	
	Agree	28(17.4)	133 (82.6)	161(82.6)	
Inappropriate consumption of antibiotics is a major cause of antibiotic resistance in this hospital	Disagree	6(42.9)	8(57.1)	147.7)	0.034^a^
	Neutral	8(26.7)	22(73.3)	3(16.6)	
	Agree	22(16.1)	115(83.9)	137(75.7)	
The easy access to antibiotics without a prescription in Ethiopia contributes to antibiotic resistance	Disagree	8(53.3)	7(46.7)	15(8.3)	0.003^a^
	Neutral	2(18.2)	9(81.8)	11(6.1)	
	Agree	26(16.8)	129(83.2)	155(85.6)	
The lack of adequate diagnostic tests in this hospital leads to overuse of antibiotics	Disagree	6(18.8)	26(81.2)	32(17.7)	0.035^b^
	Neutral	1(3.3)	29(96.7)	30(16.6)	
	Agree	29(24.4)	90(75.6(	119(65.7)	
My choice of antibiotics is often influenced by the availability of the antibiotics	Disagree	11(30.6)	25(69.4)	36(20.0)	0.012^b^
	Neutral	1(2.9)	33(97.1)	34(18.8)	
	Agree	24(21.6)	87(78.4)	111(61.3)	
I regularly consider susceptibility patterns at this hospital when empirically prescribing or recommending antibiotics	Disagree	24(29.6)	57(70.4)	81(44.8)	0.009^a^
	Neutral	5(8.9)	51(91.1)	56(30.9)	
	Agree	7(15.9)	37(84.1)	44(24.3)	
I routinely try to step down broad-spectrum antibiotics to a narrow-spectrum antibiotic after about three days	Disagree	8(12.9)	54(87.1)	62(34.3)	0.016^a^
	Neutral	17(33.3)	34(66.7)	51(28.2)	
	Agree	11(16.2)	57(83.8)	68(37.6)	
I routinely prescribe/recommend very broad-spectrum antibiotics empirically because I believe most patients are infected with a drug-resistant organism	Disagree	22(28.6)	55(71.4)	77(42.5)	0.032^a^
	Neutral	8(16.7)	40(83.3)	48(26.5)	
	Agree	6(10.7)	50(89.3)	56(30.9)	
I routinely check microbiology laboratory results to guide my choice of antibiotics	Disagree	27(31.4)	59(68.6)	86(47.5)	0.002^a^
	Neutral	6(13.6)	38(86.4)	44(24.3)	
	Agree	3(6.0)	48(94.0)	51(88.1)	
In the past seven days, I have prescribed broad spectrum antibiotics for longer than 3 days for more than 50 % of my patients	Disagree	8(11.0)	65(89.0)	73(40.3)	0.001^b^
	Neutral	9(16.1)	47(83.9)	56(30.9)	
	Agree	19(36.5)	33(63.5)	52(28.7)	
ASP has impact on an institution's nosocomial infection rates	Disagree	1(6.7)	14(99.3)	15(8.3)	0.013^a^
	Neutral	4(8.3)	44(91.7)	48(26.5)	
	Agree	31(26.3)	87(73.7)	118(65.2)	
Total for all items		36(19.9)	145(80.1)	181(100.0)	

a Hospitals attempted to implement ASP have better agreement.

b Hospitals which didn't attempted ASP do have better agreement.

Status and the current situation of ASPs in five hospitals were assessed using checklists to catch up on the availability and functionality status of ASPs, current practice in the management of infections, and the availability of resources for the implementation of ASPs. In the hospitals, there were 1718 beds, and the average bed occupancy rate was 94.5 %. On average, patients stay about five days in the hospital. There are about 18 clinical pharmacists and/or MSc holders and 9 microbiologists in the hospitals (Table 8).

**Table 8:** Distribution of health professionals in the hospitals, in 2022.

Variables		Hospitals					
		FHCSH	TGSH	DMCSH	IGH	FSGH	Total
Physicians	Internists	6	27	4	2	2	41
	Other specialization	45	101	16	7	5	174
	General Practitioners	118	100	43	33	32	326
Pharmacy professionals	Clinical pharmacist/MSc	7	1	4	2	3	18
	Pharmacist	37	36	19	7	7	105
	Other pharmacy professionals	7	20	25	7	15	74
Medical laboratory professionals	Microbiologists	2	1	4	0	2	9
	BSc+	40	42	26	9	7	124
	Others laboratory professionals	28	16	8	11	8	71
Other professionals*		459	459	415	286	192	1552
Total number of beds		800	467	216	140	95	1718
Average bed occupancy rate (%)		88	100	95.5	90	99	94.5
Average patient stays		5	7	4	5	4.7	5.14

Other professionals*: nurse, public health, environmental health, midwifery, anesthesia, HIT, optometry nurse; **Abbreviations**: FHCSH-Felege Hiwot Comprehensive Specialized Hospital; TGSH-Tibebe Ghion Specialized Hospital; DMCSH-Debre Markos Comprehensive Specialized Hospital; IGH- Injibera General Hospital; FSGH- Finote Selam General Hospital.

Although it is not functioning according to standard requirements for ASP, out of five hospitals, only three have tried ASP implementation, among which FHCSH and TGSH have an active formal ASP committee during data collection time. In DMCSH, there was a temptation to establish the committee, but currently it is not functional, and the issue of ASP has never been heard in IGH or FSGH.

In all hospitals, there was no formal procedure for all clinicians to review the appropriateness of all antibiotics within 48–72 h of initial orders (e.g., antibiotic time out), except in the form of routine morning sessions and daily rounds. There were antibiograms in FHCSH and DMCSH, although there was no formal procedure to monitor trends in AMR. The antibiograms in FHCSH were updated in a quarterly manner, but they have never been reviewed and clinically interpreted. Although there have been recent attempts to start culture and sensitivity tests in TGSH, due to a lack of supplies, there have been no antibiograms. In general hospitals, there is no laboratory facility to conduct culture and sensitivity tests. Likewise, except for attempts to evaluate vancomycin use (DMCSH and FHCSH), ceftriaxone use (FHCSH and TGSH), and customizing AWaRe classification (DMCSH and FHCSH), there was no system in place to track antibiotic use and AMR trends in all hospitals.

The ASP team in the FHCSH is represented by physician, pharmacist, infection prevention focal person, quality assurance focal person, microbiologist, nurse, and information technology professional. The same is true in TGSH, except for the inclusion of an epidemiologist than information technology professional in the team. In both cases, the team is led by internists, and the secretaries are clinical pharmacists. In FHCSH, there is an infectious disease physician, but there is none in TGSH. Out of the five hospitals, four have agreed to strengthen and/or start ASP in their hospitals. The FHCSH is ready to implement ASP in the internal medicine ward, ICU, recovery rooms, NICU, pediatrics, and OR since, according to them, these are the common sites where reserve antibiotics are commonly prescribed. Despite the lack of laboratory facilities, IGH has shown interest in implementing ASP in collaboration with Injibara University. IGH suggested internal medicine wards, pediatric wards, AICU, NICU, and surgical wards for the implementation of ASP.

C. Core Elements of ASP in Hospitals

The core elements of ASP implementation in FHRH and TGSH were reported. Only in accountability and expertise were they at least partially fulfilled. In the areas of leadership support and commitment, and action taken to implement ASP to improve antibiotic consumption, the implementation is very poor. In general, out of the seven core elements, the two hospitals nearly fulfill two components of accountability and representation of drug experts in the ASP team. But for leadership, it was 4 out of 16; action on implementing ASP and improve antibiotic consumption (8 out of 22), tracking (8 out of 18), reporting (2 out of 6), and education (1 out of 6). There was a formal, written to ASPs team members and attempts to evaluate some antibiotics, but there is no formal prospective audit or feedback for specific disease syndromes and/or antibiotics, and there is no system to improve antibiotic consumption or track antibiotic use and resistance (Table 9, Supplementary Table 2).

**Table 9:** Core elements of hospital antibiotic stewardship programs in hospitals, 2022.

Category	Questions	Responses			
		FHCSH		TGSH	
		Yes	No	Yes	No
**Leadership support and commitment**	1. Does your facility have a formal, written statement of support from leadership that supports efforts to improve antibiotic use (ASP)?	**✓**		**✓**	
	2. Does facility leadership provide stewardship program leader(s) dedicated time to manage the program and conduct daily stewardship interventions?		**✓**	**✓**	
	3. Does facility leadership provide stewardship program leader(s) any budgeted financial support including for resources (e.g., IT support, training) to effectively operate the program?		**✓**		**✓**
	4. Does your facility demonstrate leadership support for antibiotic stewardship?		**✓**		**✓**
	5. Does your ASP have a senior executive that serves as a point of contact or “champion” to help ensure the program has resources and support to accomplish its mission?	**✓**			**✓**
	6. Do stewardship program leader(s) have regularly scheduled meetings with facility leadership and/or the hospital board to report and discuss stewardship activities, resources and outcomes?		**✓**		**✓**
	7. Does your facility leadership ensure that staff from key support departments and groups has sufficient time to contribute to stewardship activities?		**✓**		**✓**
	8. Does facility leadership ensure that ASP activities are integrated into other quality improvement and patient safety efforts, such as sepsis management and diagnostic stewardship?		**✓**		**✓**
**Accountability**	9. Does your facility have a leader or co-leaders responsible for program management and outcomes of stewardship activities?	**✓**		**✓**	
	10. Is there a pharmacist or microbiologist or infectious disease specialist, leader responsible for working to improve antibiotic consumption for working to improve antibiotic use at your facility?	**✓**		**✓**	
	11. Does your facility have a pharmacist member/leader responsible for program outcomes of stewardship activities at your facility?	**✓**		**✓**	
**Drug Expertise**	13. Does your facility have a pharmacist(s) responsible for leading implementation efforts to improve antibiotic use?	**✓**		**✓**	
	14. Do your pharmacist(s) leading implementation efforts have specific training and/or experience in ASP?	**✓**		**✓**	
	15. Does your facility have access to individual(s) with ASP expertise?		**✓**		**✓**
**Action: Implement ASP And improve antibiotic consumption**	16. Does your facility perform prospective audit and feedback for specific antibiotic agents?		**✓**		**✓**
	17. Does your facility perform preauthorization for specific antibiotic agents?	**✓**			**✓**
	18. Does your facility have facility-specific treatment recommendations, based on national guidelines and local pathogen susceptibilities, to assist with antibiotic selection for common clinical conditions?		**✓**	**✓**	
	19. Does your facility have specific interventions to ensure optimal use of antibiotics for treating the most common infections in most hospitals?		**✓**	**✓**	
	20. Does your facility have specific interventions in place to ensure optimal use of antibiotics in specific infection/disease situations?		**✓**	**✓**	
	21. Does your facility have a policy that requires prescribers to document in the medical record or during order entry a dose, duration and indication for all antibiotic prescriptions?	**✓**		**✓**	
	22. Does your facility have a formal procedure for all prescribers to conduct daily reviews of antibiotic selection until a definitive diagnosis and treatment duration are established?		**✓**		**✓**
	23. Does your ASP assess how often patients are discharged on the correct antibiotics for the recommended duration?		**✓**		**✓**
	24. Does your ASP track antibiotic resistance?		**✓**		**✓**
	25. Does your facility have policies to improve antibiotic prescribing/use?		**✓**		**✓**
	26. Does your facility implemented practices to improve antibiotic consumption?		**✓**		**✓**
**Action:** Tracking	27. Does your ASP track antibiotic use?	**✓**		**✓**	
	28. Does your ASP monitor prospective audit and feedback interventions by tracking the types of interventions and acceptance of recommendations?		**✓**	**✓**	
	29. Does your ASP monitor preauthorization interventions by tracking which agents are being requested for which conditions?	**✓**			**✓**
	30. Does your ASP monitor adherence to facility specific treatment recommendations?		**✓**		**✓**
	31. Does your ASP monitor adherence to a documentation policy (dose, duration and indication)?		**✓**	**✓**	
	32. Does your ASP monitor the performance of antibiotic timeouts to see how often these are being done and if opportunities to improve use are being acted on during timeouts?		**✓**		**✓**
	33. Does your ASP routinely perform medication use evaluations to assess courses of therapy for select antibiotics and/or infections to identify opportunities to improve use?	**✓**		**✓**	
	34. Does your ASP routinely perform medication use evaluations to assess courses of therapy for select antibiotics and/or infections to identify opportunities to improve use?	**✓**		**✓**	
	35. Does your ASP assess how often patients are discharged on the correct antibiotics for the recommended duration?		**✓**		**✓**
	36. Does pharmacist/s support ASP activities?	**✓**		**✓**	
	37. Does your facility monitor one or more measures of antibiotic consumption?	**✓**		**✓**	
	38 Does your facility monitor one or more outcomes of antibiotic consumption?		**✓**		**✓**
**Reporting**	39. Does your ASP share facility and/or individual prescriber-specific reports on antibiotic use with prescribers?		**✓**		**✓**
	40. Does your ASP report adherence to treatment recommendations to prescribers?	**✓**		**✓**	
	41. Has your facility distributed a current antibiogram to prescribers within 18–24 months?		**✓**		**✓**
**Education**	42. Does your ASP provide education to prescribers and other relevant staff on optimal prescribing, adverse reactions from antibiotics, and antibiotic resistance?		**✓**		**✓**
	43. Does your ASP provide education to prescribers as part of the prospective audit and feedback process?		**✓**		**✓**
	44. Does your ASP provide educational resources/materials on antibiotic resistance and opportunity to improve antibiotic consumption?		**✓**	**✓**	
**Additional Questions on ASP Challenges**	45. Are there areas of antibiotic misuse in your facility?	**✓**		**✓**	
	46. Are there barriers to starting the ASP?		**✓**		**✓**

**Abbreviations:** FHCSH-Felege Hiwot Comprehensive Specialized Hospital; TGSH-Tibebe Ghion Specialized Hospital.

## 4 Discussion

AMR is a public health problem globally [1,35,[56], [57], [58], [59]] and in Ethiopia [13,[60], [61], [62], [63]]. Implementation of agreed-upon ASPs, which do have beneficial clinical and economic impacts, following identified gaps is a key to tackle AMR [64,65]. Identifying perceptions, enablers, and barriers to ASP implementation is a critical step for the successful implementation of ASPs in hospitals [44,[66], [67], [68], [69], [70]]. Determining the knowledge, perceptions and attitudes of hospital-based clinicians toward antimicrobial prescription, AMR, and ASP, including the types of activities implemented in hospital-based ASPs, the methods used to measure the impact of ASP, and factors influencing the antimicrobial prescribing behavior of physicians, are necessary components to initiating ASP [71]. Thus, this study assessed the readiness of hospitals for implementation of ASP, explored physicians' and pharmacists’ perceptions about AMR, antibiotic prescribing or prescribing practices, ASP, and suggested potential interventions to combat AMR in the hospitals. The key findings will be discussed hereafter.

In the current study, 163 (90.1 %), 161 (89.0 %) and 128 (70.7 %) of the health professionals believed that AMR was a significant problem both in Ethiopia, worldwide, and in their hospitals respectively. Although there was a slight difference on magnitude, the report was almost similar to a report on health professionals' beliefs at Black Lion Hospital, which showed that antibiotic resistance is a significant problem worldwide (94.6 %), in Ethiopia (91.7 %) and in their hospital (84.5 %) [44], 93 % of physicians in Cameron perceive that AMR is a nationwide problem [72], physicians in Nigeria recognize AMR as a global (95.4 %) and local problem (81.1 %) [73], and in another study in Nigeria among health professionals, 96.0 % of whom believed that antibiotic resistance was a problem in Nigeria, 84.0 % agreed that AMR was a problem for their practice [46] but in Ghana very few physicians perceived antibiotic resistance as global problem (30.1 %), national problem (18.5 %), and problem in their hospital (8.1 %) [74]. In South Africa, 78.5 % of pharmacists perceived that AMR was a problem in their hospitals [75], and in Zambia, 95 % of health professionals perceived AMR as a current problem in their practice [76]. The current study also revealed a difference in level of agreement among physicians and pharmacy professionals in believing that a very high proportion (>30 %) of gram-negative infections are highly drug-resistant (resistant to all cephalosporins and some are even resistant to carbapenems) and that a very high proportion (>30 %) of staphylococcal infections are resistant to methicillin, which was more among pharmacy professionals, which was different from a report which didn't show any significant difference between physicians and pharmacy professionals [44]. The difference in reported magnitude might be attributed to differences in services and experience with AMR in hospitals in different countries and experiences in ASP implementation.

In the current study, health professionals believed that easy access to antibiotics without a prescription 155 (85.6 %), inappropriate consumption of antibiotics 137 (75.7 %), lack of adequate surveillance for drug-resistant organisms 127 (70.2 %) and adequate staff education regarding antibiotic consumption and resistance 124 (68.5 %), and overuse of antibiotics due to a lack of diagnostic tests 119(65.7 %) were perceived as key contributors to AMR in hospitals. This was similar to the study in Black Lion Hospital, which reported 84.3 % agreement with easy access to antibiotics, 82.0 % inappropriate consumption, and 64.4 % lack of adequate diagnostic tests as the main perceived causes of AMR [44] and in Ghana overuse of antibiotics in hospitals (67.5 %), uncompleted antibiotic therapy were common causes for AMR (50.31 %) and poor quality of antibiotics (37.7 %) [74]. In Uganda, antimicrobial overuse at hospitals (91 %), poor infection control practices by health professionals (79 %), and prescribing broad-spectrum antimicrobials when equally effective narrower-spectrum antimicrobials are available (73 %), were perceived causes for AMR [70], and in Cameron, physicians perceived that inappropriate use of antibiotics (94 %), overuse of antibiotics (87 %), and indiscriminate use of broad-spectrum antibiotics (71 %), were possible causes for AMR [72]. The current study also revealed perceived difference between pharmacy professionals and physicians on the causes of AMR. This was similar to a study at Black Lion hospital [44]. Overall, the perceived causes assures the general agreement that inappropriate prescribing is a key contributory factor to AMR [35,36] which occur in up to 50 % of in hospital prescriptions [41]. This supports the fact that AMR is a major global public health concern, with serious consequences in LMICs which is due to widespread and inappropriate use of antibiotics in along with inadequate management of pharmaceutical wastes [20].

In this study, health professionals uncovered their intention to prescribe/dispense antibiotics to 86 (47.5 %) without checking microbiology laboratory results to guide their choice of antibiotics, lab results were not communicated timely if available to 84 (46.4 %) and antibiotic susceptibility patterns were not considered when prescribing antibiotics empirically in 81 (44.8 %). This was similar to health professionals belief that microbiology laboratory were not communicated in a timely manner (85 %), and prescribing/dispensing antibiotics without checking laboratory results to guide the choice of therapy (65 %) in Black Lion Hospital [44], and perceived factors of lack of laboratory tests (93.9 %), availability of antibiotics (80.6 %), and cost of antibiotics (80.6 %) influencing empiric therapy in Nigeria [77], although there are differences in magnitude which were lower in the current study (cost antibiotics 126 (69.6 %), availability of antibiotics 111 (61.3 %) and a lack of diagnostic tests 119 (65.7 %)). The findings confirmed the claim that antibiotic prescribing in Africa is highly prevalent, ranging from 38 % to 80 % (average 50 %), and intravenous to oral switch and documentation of start and stop dates for antibiotics were the least reported [64]. The current study also revealed the difference in perception between physicians and pharmacists in prescribing/dispensing of antibiotics, which is similar to reports at Black Lion Hospital in Ethiopia [44].

In the current study, health professionals believed that ASP reduces the duration of hospital stays and associated costs 137 (75.7 %), improves the quality of patient care 133(73.5 %), reduces overall antibiotic consumption and can result in cost savings 129(71.3 %), and reduces the problem of AMR 126(69.6 %). Almost similar results were reported from Black Lion Hospital with a slightly higher magnitude, where 82.4 % believed that ASP improves the quality of patient care, ASP reduces overall antibiotic consumption and can result in cost savings (76.9 %), ASP reduces the duration of hospital stay and associated costs (73.5 %), and ASP reduces the problem of AMR (82.9 %) [44]. In Libya, pharmacy professionals believed that ASP reduces the rate of AMR (70.2 %), reduces inappropriate use of antibiotics in the community (68.4 %), participation in ASP activities will boost public confidence in community pharmacy services (79 %), community pharmacists’ participation in the ASP will promote (73.7 %) and ASP activities are important in the hospital setting but are not important in the community setting (16.7 %) [78]. This assures that reducing AMR is a public health priority [58], and the implementation of ASP reduces inappropriate antibiotic use and AMR in hospital settings, in the community and private health facilities as well [4,51,[78], [79], [80]].

The current study identified a lack of funding, infectious diseases physicians and trained pharmacists on ASPs, inadequate information technology resources, a lack of incentives and support for committees, a lack of documentation and reporting mechanisms, inadequate initiatives for antimicrobial control and management, and a lack of microbiology laboratories and reagents in most of the hospitals. In Sri Lanka, Kenya, and Tanzania, the barriers to implementing ASP were expensive antimicrobials, limited antimicrobial availability, resistance to changing current practices regarding antimicrobial prescribing, and limited diagnostic capabilities [68], whereas in Nigeria there was a lack of funding, poor awareness about ASP, prescribers’ opposition, a lack of IT facilities, the unavailability of an ASP committee, a lack of trained staff, a lack of leadership support and administration not being aware of ASPs [81]. The challenges for ASP implementation in a resource-limited setting are lack of financing, access to technologies, and capacity building [82]. Access to formalized infectious diseases or clinical microbiology support, lack of access to education and training, and lack of internal expertise within healthcare facilities, especially pharmacists with ASP skills, are also common barriers to the implementation of ASP in hospitals [83]. Thus, the successful implementation of ASPs needs targeted diagnosis and treatment, developing and updating antibiograms to direct empiric antimicrobial use, and increasing the use of microbiologic tests [1,84].

The current study uncovered the status of ASPs in hospitals and gaps in implementation of the CDC's Core Elements of ASP in hospitals, which attempted to implement ASP, which is known to ensure appropriate use of antibiotics [1]. The hospitals didn't implement any specific ASP strategy or set metrics to evaluate the ASPs, except for attempts to evaluate vancomycin use, ceftriaxone use, and customizing the WHO AWaRe classification for antibiotics. This supports the lack of budget lines for AMS activities in 23 (74.2 %) countries in the WHO African region, where only 25.8 % of the countries had developed a national ASP implementation policy incorporating defined goals, targets, and operational plans [85]. Similar study in Nigeria reported lack of ASPs in a substantive proportion of hospitals, and suboptimal functioning in those who attempted to implement ASPs [86]. Successful ASP implementation relies on a strong commitment from senior hospital leadership, accountability, and responsibilities, among other core elements [87], but leadership commitment, action on implementing ASP and improving antibiotic consumption, tracking and reporting, and education were very poor in the current study. The presence of formal multidisciplinary organizational team structures responsible for ASP is one opportunity to strengthen the existing ASPs, as evidenced by the 79 % increase and improvements pertained to the core ASP checklist element on the multidisciplinary organizational structures responsible for ASP in Tanzania, Zambia, Uganda, and Ghana after ASP implementation [69]. Different strategies, such as prescriber education, formulary restriction, prior approval, streamlining, antibiotic cycling, and computer-assisted programs, have been proposed to improve antibiotic use [4,88]. Attempts for the WHO AWaRe categorization of antibiotics, which is an initiative to facilitate ASP, taking into consideration the potential of different antibiotic classes to cause resistance when used for the treatment of clinical infections [85] and evaluation of Watch antibiotics (vancomycin and ceftriaxone) as an early detection for inappropriate antibiotic use and signals the need for intervention [2,3,85] is an opportunity to further strengthen the ASP in the hospitals.

In resource-limited settings, a lack of clear political commitment, overcrowded healthcare systems, inadequate funding, lax legal and regulatory frameworks, the absence of electronic health record systems, non-uniform access to diagnostics, issues with access to quality-assured medicines, limited knowledge and awareness, and a shortage of trained manpower are challenges for the successful implementation of the core elements of ASP [88]. There are marked gaps and variability in the implementation and maturity of NAPs to facilitate sustainable delivery and operationalization at country and regional levels and to identify AMR-related priorities that are relevant to infrastructure needs and contexts [30]. Thus, the design and implementation of ASP interventions should foster data management and sharing tools between physicians, pharmacists, and microbiology laboratories using antibiotic prescribing guidelines based on hospital epidemiology data and easily accessible antibiograms [89,90]. Of course, political will from governments is critical in establishing, implementing, and sustaining ASPs [91].

In many LMICs, including Sub-Saharan Africa, pharmacist-led antimicrobial stewardship program interventions were shown to improve adherence to guidelines, reduce inappropriate prescribing, and improve compliance with process measures [10,92,93]. In this hospital, the availability of specialized physicians and laboratory technologists is an additional opportunity to start and strengthen ASP in hospitals. Although the role of the ASP pharmacist in the preservation of antibiotics through the monitoring, evaluation, and guidance of appropriate antibiotic use is vast [94], cooperation between physicians and pharmacists is essential to ensure high-quality treatment and improve patient outcomes [95]. Due to the complexity and challenges of implementing various ASP strategies in LIMCs, it is advised to rely on the plan-do-check-act (PDCA) model to improve efficiency and the likelihood of success of the programs through faster troubleshooting and continuous maintenance in the system [90]. The positive perception of health professionals towards hospital-based ASPs and potential interventions will help ensure the successful implementation of quality improvement ASPs in hospitals. Since the success of ASPs will be hampered by a lack of laboratory and technological support, limited stewardship time, poor documentation, and a lack of guidelines and policies [92], to achieve better outcomes from the interventions (antibiotic use, patient outcomes, and economic outcomes) [65], examining use practices, documenting antibiotic use data, and serving as an educational resource for clients, as well as strengthening and establishing diagnostic labs to provide antibiograms, is critical [1].

It was believed that education on antibiotic therapy for medical and pharmacy staff 151(83.4 %), developing new institutional guidelines for empiric antibiotic use 143(79.0 %), implementation of prospective audits and feedback 142(78.5 %), active involvement of hospital infection prevention and control teams 137(75.7 %) and access to institution-specific antibiograms to treating teams 137(75.5 %) were believed to be effective interventions. This was similar to the report at Black Lion Hospital, which ranges from 92.9 % for education to 83.2 % each for active involvement of hospital infection prevention and control teams and access to institution-specific antibiogram to treating teams [44]. These strategies were included in the national ASP guideline for Ethiopia [3]. Together with other strategies, education is a key component of every coordinated ASP effort to optimize hospital antimicrobial use due to its direct impact on healthcare professionals’ knowledge, attitude, and practice [80]. As has been evidenced, even attempts to implement ASPs do have a positive impact on the perception of professionals to contain AMR and implement ASP.

The major findings from this study were most respondents do have positive perception towards implementation of ASP where pharmacists had more positive concerns towards AMR, causes for AMR and implementation of ASP in the hospitals. It also documents availability of ASP teams in some of the hospitals and attempts to document WHO AWaRe categorization and evaluations of most commonly consumed of Watch antibiotics in the hospitals. Some limitations in this study must be noted. The study didn't collect qualitative data based on key informant interviews and focus group discussions but simply collected quantitative data and assessed the status of hospitals based on checklists. The sampling was not done systematically but rather collected data from available staff during the data collection period. Since questionnaires were distributed and collected back, a potential bias would have been introduced if the participants had discussed them with their colleagues while filling them out. The hospitals that didn't attempt ASP were not included in the core elements analysis but rather were included in the readiness assessment. We didn't validate the core elements of ASPs filled by medical directors or leaders of ASPs. Since the findings were based on five hospitals, they may not be generalizable to other hospitals.

In conclusion, the status of ASP in hospitals is very poor. Although it is not of concern in general hospitals, the issue of ASP is, in principle, accepted in comprehensive specialized hospitals and referral hospitals. AMR was perceived as a problem in at the global level and in Ethiopia, where easy access to antibiotics without a prescription, inappropriate consumption of antibiotics, and overuse of antibiotics were perceived as key contributors to AMR in hospitals. Antibiotics were believed to be prescribed/dispensed without laboratory results, and antibiotic susceptibility patterns were not considered to prescribing antibiotics empirically. To tackle the problems, pharmacist-led prospective audits and feedback with education and institutional guidelines for empiric antibiotic use can be better implemented in the hospitals. Involvement of hospital infection prevention and control teams and collaboration between hospitals in ASP implementation help establish a strong ASP in the area.

## Data availability

The data associated with this study is not deposited into a publicly available repository but all data are included in the manuscript. We will avail additional data if any when requested.

## CRediT authorship contribution statement

**Asrat Agalu Abejew:** Writing – review & editing, Writing – original draft, Visualization, Supervision, Software, Methodology, Formal analysis, Data curation, Conceptualization. **Gizachew Yismaw Wubetu:** Writing – review & editing, Supervision, Conceptualization. **Teferi Gedif Fenta:** Writing – review & editing, Supervision, Conceptualization.

## Declaration of competing interest

The authors declare the following financial interests/personal relationships which may be considered as potential competing interests:

Asrat Agalu reports financial support provided by Addis Ababa University College of Health Sciences. Other authors, has declared that they have no known competing financial interests or personal relationships that could have appeared to influence the work reported in this paper.
