# Supplementary material for: Assessment of challenges and opportunities in antibiotic stewardship program implementation in Northwest Ethiopia
Source: Heliyon. 2024 Jun 7;10(11):e32663. doi: 10.1016/j.heliyon.2024.e32663 (PMC11193037; doi:10.1016/j.heliyon.2024.e32663)
Supplement: Multimedia component 1 [file mmc1.docx]

**Supplementary table 1**:  Belief of health professionals on Scope and factors contributing to antibiotic resistance in the hospitals, 2013 (N=181)

| Statements | Frequency (%) of responses on each statement | | | | | | | |
| --- | --- | --- | --- | --- | --- | --- | --- | --- |
|  | Strongly Agree | Agree | Neutral | Disagree | Strongly Disagree | NA | Mean | |
| **Scope of antibiotic resistance problem** | | | | | | | | |
| AMR is a significant problem worldwide | 118(65.2) | 44(24.3) | 6(3.3) | 4(2.2) | 9(5) | 0 | | 4.43 |
| AMR is a significant problem in my country | 107(59.1) | 53(29.3) | 7(3.9) | 7(3.9) | 6(3.3) | 1(0.6) | | 4.39 |
| AMR is a significant problem in my hospital | 73(40.3) | 54(29.8) | 41(22.7) | 5(2.8) | 6(3.3) | 2(1.1) | | 4.04 |
| AMR is a problem in my daily practice | 42(23.2) | 65(35.9) | 49(27.1) | 12(6.6) | 9(5) | 4(2.2) | | 3.72 |
| A patient is likely to develop drug-resistant infection during their hospital stay at this hospital | 32(17.7) | 76(42) | 42(23.2) | 22(12.2) | 8(4.4) | 1(0.6) | | 3.58 |
| I think a very high proportion (>30%) of gram-negative infections are highly drug- resistant in this hospital (resistant to all cephalosporins, and some are even resistant to carbapenems) | 14(7.7) | 38(21) | 87(48.1) | 17(9.4) | 10(5.5) | 15(8.3) | | 3.41 |
| I think a very high proportion (>30%) of Staphylococcal infections are resistant to methicillin (MRSA) in this hospital | 16(8.8) | 37(20.4) | 76(42) | 26(14.4) | 7(3.9) | 19(10.5) | | 3.48 |
| **Beliefs on factors contributing to antibiotic resistance in the hospitals** | | | | | | | | |
| Inappropriate consumption of antibiotics is a major cause of antibiotic resistance in this hospital | 76(42) | 61(33.7) | 29(16) | 10(5.5) | 4(2.2) | 1(0.6) | | 4.09 |
| The easy access to antibiotics without a prescription in Ethiopia contributes to antibiotic resistance | 112(61.9) | 43(23.8) | 10(5.5) | 6(3.3) | 9(5) | 1(0.6) | | 4.36 |
| The prescription of broad-spectrum antibiotics is directly linked to antibiotic resistance in this hospital | 50(27.6) | 54(29.8) | 46(25.4) | 13(7.2) | 16(8.8) | 2(1.1) | | 3.64 |
| This hospital performs adequate surveillance for drug-resistant organisms | 5(2.8) | 14(7.7) | 31(17.1) | 60(33.1) | 67(37) | 4(2.2) | | 2.13 |
| The lack of adequate diagnostic tests in this hospital leads to overuse of antibiotics | 63(34.8) | 56(30.9 | 30(16.6) | 19(10.5) | 11(6.1) | 2(2.2) | | 4.32 |
| This hospital provides adequate staff education regarding antibiotic consumption and resistance | 12(6.6) | 11(6.1) | 33(18.2) | 62(34.3) | 61(33.7) | 2(2.2) | | 2.21 |
| I suspect that antibiotics available in my hospital are of poor quality and might not be effective | 10(5.5) | 17(9.4) | 50(27.6) | 60(33.1) | 41(22.7) | 3(1.7) | | 2.47 |
| The sporadic supply of antibiotics in my hospital leads to interruptions of therapy thereby contributing to antibiotic resistance | 28(15.5) | 62(34.3) | 50(27.6) | 27(14.9) | 13(7.2) | 1(0.6) | | 3.38 |
| The lack of close clinical follow-up during antibiotic consumption in my hospital contributes to antibiotic resistance | 38(21) | 67(37) | 40(22.1) | 26(14.4) | 8(4.4) | 2(1.1) | | 3.58 |
| Patient demands and expectations contribute to overuse of antibiotics in this hospital | 20(11) | 48(26.5) | 58(32) | 33(18.2) | 20(11) | 2(1.1) | | 3.12 |
| Poor infection control practices by health professionals significantly contributes to the spread of antibiotic resistance in this hospital | 30(16.6) | 75(41.4) | 39(21.5) | 27(14.9) | 9(5) | 1(0.6) | | 3.51 |
| Adherence to hand-hygiene protocols is acceptable at this hospital | 23(12.7) | 41(22.7) | 48(26.5) | 44(24.3) | 25(13.8) | 0 | | 2.96 |
| Patient rooms and equipment are cleaned appropriately as per hospital cleaning protocol once a patient carrying a drug-resistant organism (DRO) has been discharged from this hospital | 11(6.1) | 27(14.9) | 49(27.1) | 51(28.2) | 39(21.5) | 4(2.2) | | 2.62 |
| **Antibiotics prescription/dispensing practices** | | | | | | | | |
| My choice of antibiotics is often influenced by the availability of the antibiotics rather than by the local antibiogram or by the etiologic cause of disease (availability of laboratory results) | 39(21.5) | 72(39.8) | 33(18.2) | 18(9.9) | 18(9.9) | 1(0.6) | | 3.55 |
| Cost considerations for the patient affects my choice of antibiotics | 41(22.7) | 83(45.9) | 26(14.4) | 12(6.6) | 17(9.4) | 2(1.1) | | 3.66 |
| Microbiology lab results are timely communicated to the health professionals in this hospital | 9(5) | 32(17.7) | 43(23.8) | 41(22.7) | 43(23.8) | 13(7.2) | | 2.79 |
| I regularly refer to/consider the antibiotic susceptibility patterns at this hospital (institutional antibiogram) when empirically prescribing or recommending antibiotics | 5(2.8) | 39(21.5) | 46(25.4) | 44(24.3) | 37(20.4) | 10(5.5) | | 2.78 |
| If medically appropriate, I routinely try to step down intravenous antibiotics to an oral alternative antibiotic after about three days | 33(18.2) | 71(39.2) | 38(21) | 28(15.5) | 11(6.1) | 0 | | 3.48 |
| If medically appropriate, I routinely try to step down broad-spectrum antibiotics to a narrow-spectrum antibiotic after about three days | 24(13.3) | 44(24.3) | 50(27.6) | 40(22.1) | 22(12.2) | 1(0.6) | | 3.06 |
| Restrictions on antibiotics could impair my ability to provide good patient care | 19(10.5) | 60(33.1) | 38(21) | 42(23.2) | 22(12.2) | 0 | | 3.07 |
| I routinely prescribe/recommend very broad-spectrum antibiotics empirically because I believe most patients are infected with a drug-resistant organism | 12(6.6) | 44(24.3) | 43(23.8) | 49(27.1) | 28(15.5) | 5(2.8) | | 2.88 |
| I routinely prescribe/recommend very broad-spectrum antibiotics empirically because microbiology lab results are not available in a timely fashion | 46(25.4) | 55(30.4) | 18(9.9) | 39(21.5) | 17(9.4) | 6(3.3) | | 3.51 |
| I routinely check microbiology laboratory results to guide my choice of antibiotics | 10(5.5) | 40(22.1) | 35(19.3) | 47(26) | 39(21.5) | 10(5.5) | | 2.81 |
| In the past seven days, I have prescribed broad spectrum antibiotics for longer than 3 days for fewer than 10% of my patients; | 13(7.2) | 38(21) | 50(27.6) | 44(24.3) | 26(14.4) | 10(5.5) | | 2.99 |
| In the past seven days, I have prescribed broad spectrum antibiotics for longer than 3 days for 10-50% of my patients | 14(7.7) | 38(21) | 47(26) | 45(24.9) | 24(13.2) | 13(7.2) | | 3.07 |
| In the past seven days, I have prescribed broad spectrum antibiotics for longer than 3 days for more than 50% of my patients | 16(8.8) | 36(19.9) | 43(23.8) | 47(26) | 26(14.4) | 13(7.2) | | 3.04 |
| **Perception on Implementation of ASP** | | | | | | | | |
| ASPs (ASP) improve quality of patient care | 70(38.7) | 63(34.8) | 29(16) | 2(1.1) | 7(3.9) | 10(5.5) | | 4.20 |
| ASP reduces overall antibiotic consumption and can result in cost savings | 61(33.7) | 68(37.6) | 31(17.1) | 10(5.5) | 5(2.8) | 6(3.3) | | 4.04 |
| ASP reduces duration of hospital stay and associated costs | 52(28.7) | 85(47) | 19(10.5) | 13(7.2) | 7(3.9) | 5(2.8) | | 3.98 |
| ASP reduce the problem of antibiotic resistance | 61(33.7) | 65(35.9) | 29(16) | 13(7.2) | 7(3.9) | 6(3.3) | | 3.98 |
| ASP has impact on an institution’s nosocomial infection rates | 39(21.5) | 79(43.6) | 41(22.7) | 9(5) | 6(3.3) | 7(3.9) | | 3.87 |
| This hospital has the capacity to establish and implement an effective ASP | 35(19.3) | 58(32) | 47(26) | 24(13.3) | 11(6.1) | 6(3.3) | | 3.55 |
| My individual efforts in antibiotic stewardship can significantly impact this hospital’s antibiotic resistance problem | 37(20.4) | 68(37.6) | 46(25.4) | 19(10.5) | 7(3.9) | 4(2.2) | | 3.67 |
| I would like to receive more feedback on my antibiotic selection | 37(20.4) | 84(46.4) | 31(17.1) | 20(11) | 4(2.2) | 5(2.8) | | 3.80 |
| ASPs can be an obstacle to good patient care | 12(6.6) | 17(9.4) | 38(21) | 60(33.1) | 51(28.2) | 3(1.7) | | 2.38 |
| ASPs override prescribers’ decision autonomy | 10(5.5) | 27(14.9) | 57(31.5) | 56(30.9) | 27(14.9) | 4(2.2) | | 2.72 |
| I do not have enough time to further invest into ASP | 7(3.9) | 23(12.7) | 50(27.6) | 66(36.5) | 29(16) | 6(3.3) | | 2.62 |
| Infectious diseases experts that can provide guidance in antibiotic selection and prescription are available in this hospital | 21(11.6) | 27(14.9) | 39(21.5) | 54(29.8) | 36(19.9) | 4(2.2) | | 2.75 |
| Additional staff education on antibiotic prescribing and use is needed in this hospital | 64(35.4) | 59(32.6) | 26(14.4) | 19(10.5) | 12(6.6) | 1(0.6) | | 3.81 |
| Prescribing physicians are the only professionals who need to understand antibiotic stewardship | 20(11) | 19(10.5) | 15(8.3) | 54(29.8) | 72(39.8) | 1(0.6) | | 2.25 |
| Pharmacists with sufficient training to provide guidance on antibiotics (ex. Antibiotic switches, IV to PO step-down, renal dose adjustments) are available in this hospital | 17(9.4) | 36(19.9) | 36(19.9) | 39(21.5) | 47(26) | 6(3.3) | | 2.75 |
| Implementation of electronic medical recording (e.g., receiving results electronically) can improve effect of ASP | 42(23.2) | 78(43.1) | 45(24.9) | 7(3.9) | 7(3.9) | 2(1.1) | | 3.81 |
| **Belief on potential intervention to combat AMR** | Effective | Unsure | Not useful | Mean |  | | | |
| Education on antibiotic therapy to medical and pharmacy staff | 151(83.4) | 18(9.9) | 12(6.6) | 2.768 |  |  |  |  |
| Develop new institutional guidelines for empiric antibiotic use | 143(79) | 22(12.2) | 16(8.8) | 2.7017 |  |  |  |  |
| Access to institution-specific antibiogram to treating teams | 137(75.5) | 30(16.6) | 14(7.7z0 | 2.6796 |  |  |  |  |
| Implementation of prospective audit and feedback (multidisciplinary rounds on appropriate prescribing and use of antibiotics) | 142(78.5) | 29(16) | 10(5.5) | 2.7293 |  |  |  |  |
| Active involvement of hospital infection prevention and control team | 137(75.7) | 31(17.1) | 13(7.2) | 2.6851 |  |  |  |  |
| Antibiotic cycling intervention (e.g., scheduled rotation of 3rd or 4th generation cephalosporins with carbapenems and piperacillin-tazobactam for pre-determined time periods) | 107(59.2) | 64(35.4) | 10(5.5) | 2.5525 |  |  |  |  |
| Antibiotic restriction intervention (certain antibiotics cannot be prescribed without infectious disease specialist approval for restricted antibiotics) | 108(59.7) | 42(23.2) | 31(17.1) | 2.4254 |  |  |  |  |

**Supplementary table 2**: Core Elements of Hospital Antibiotic Stewardship Programs in two Hospitals, 2022

| **Category** | Questions | **Response** | | | |
| --- | --- | --- | --- | --- | --- |
|  |  | **FHCSH** | | **TGSH** | |
|  |  | **Yes** | **No** | **Yes** | **No** |
| **Leadership support and commitment** | 1. Does your facility have a formal, written statement of support from leadership that supports efforts to improve antibiotic use (ASP)? | **√** |  | **√** |  |
|  | 2.  Does facility leadership provide stewardship program leader(s) dedicated time to manage the program and conduct daily stewardship interventions? |  | **√** | **√** |  |
|  | 3. Does facility leadership provide stewardship program leader(s) any budgeted financial support including for resources (e.g, IT support, training) to effectively operate the program? |  | **√** |  | **√** |
|  | 4. Does your facility demonstrate leadership support for antibiotic stewardship? If yes, indicate which of the following are in place (select all that apply) |  | **√** |  | **√** |
|  | -Antibiotic stewardship duties are included in medical director, nursing and or pharmacy job description |  | **√** |  | **√** |
|  | -Leadership monitors adherence to antibiotic stewardship policies |  | **√** | **√** |  |
|  | -Antibiotic consumption and resistance data are reported in the quality assessment and assurance (QAA) meetings |  | **√** |  | **√** |
|  | 5. Does your antibiotic stewardship program have a senior executive that serves as a point of contact or “champion” to help ensure the program has resources and support to accomplish its mission? | **√** |  |  | **√** |
|  | 6.Do stewardship program leader(s) have regularly scheduled meetings with facility leadership and/or the hospital board to report and discuss stewardship activities, resources and outcomes? |  | **√** |  | **√** |
|  | 7. Does your facility leadership ensure that staff from key support departments and groups has sufficient time to contribute to stewardship activities? |  | **√** |  | **√** |
|  | 8.Does facility leadership ensure that antibiotic stewardship activities are integrated into other quality improvement and patient safety efforts, such as sepsis management and diagnostic stewardship? |  | **√** |  | **√** |
| **Accountability** | 9.Does your facility have a leader or co-leaders responsible for program management and outcomes of stewardship activities? | **√** |  | **√** |  |
|  | 10.Is there a pharmacist or microbiologist or infectious disease specialist, leader responsible for working to improve antibiotic consumption for working to improve antibiotic use at your facility? | **√** |  | **√** |  |
|  | 11.Does your facility have a pharmacist member/leader responsible for program outcomes of stewardship activities at your facility? | **√** |  | **√** |  |
| **Drug Expertise** | 13.Does your facility have a pharmacist(s) responsible for leading implementation efforts to improve antibiotic use? | **√** |  | **√** |  |
|  | 14.Do your pharmacist(s) leading implementation efforts have specific training and/or experience in antibiotic stewardship? | **√** |  | **√** |  |
|  | 15.Does your facility have access to individual(s) with antibiotic stewardship expertise? |  | **√** |  | **√** |
|  | If yes, indicate who serves as your antibiotic stewardship expert (select all that apply) |  |  |  | **√** |
|  | - Other staffs work with the stewardship teams to improve antibiotic use (Check all that applies) | **√** |  |  | **√** |
|  | A. Infection Prevention and Healthcare Epidemiology | **√** |  |  |  |
|  | B. Quality assurance | **√** |  |  |  |
|  | C. Microbiology (Laboratory) | **√** |  |  |  |
|  | D. Nursing | **√** |  |  |  |
|  | E. Information Technology (IT) | **√** |  |  |  |
| **Action: Implement** | 16.Does your facility perform prospective audit and feedback for specific antibiotic agents? | **√** |  | **√** |  |
|  | 17. Does your facility perform preauthorization for specific antibiotic agents? | **√** |  |  | **√** |
|  | 18.Does your facility have facility-specific treatment recommendations, based on national guidelines and local pathogen susceptibilities, to assist with antibiotic selection for common clinical conditions? |  | **√** | **√** |  |
|  | 19.Does your facility have specific interventions (e.g., ensuring correct discharge duration of therapy) to ensure optimal use of antibiotics for treating the most common infections in most hospitals? |  | **√** | **√** |  |
|  | 20. Does your facility have specific interventions in place to ensure optimal use of antibiotics in specific infection/ disease situations? |  | **√** | **√** |  |
|  | 21. Does your facility have a policy that requires prescribers to document in the medical record or during order entry a dose, duration and indication for all antibiotic prescriptions? | **√** |  | **√** |  |
|  | 22. Does your facility have a formal procedure for all prescribers to conduct daily reviews of antibiotic selection until a definitive diagnosis and treatment duration are established (i.e. time out)? |  | **√** |  | **√** |
| **Action: Improve Antibiotic consumption** | 23.Does your antibiotic stewardship program assess how often patients are discharged on the correct antibiotics for the recommended duration? |  | **√** |  | **√** |
|  | 24.Does your antibiotic stewardship program track antibiotic resistance? |  | **√** | **√** |  |
|  | 25.Does your facility have policies to improve antibiotic prescribing/use? If yes, indicate which policy is currently active (select all that apply) | **√** |  |  | **√** |
|  | -All antibiotic orders must have dose, frequency, duration, and indication | **√** |  | **√** |  |
|  | -Use of facility-specific algorithms to assess residents for suspected infections |  | **√** |  | **√** |
|  | -Use of facility-specific algorithms to request diagnostic tests for specific infections |  | **√** |  | **√** |
|  | -Use of facility-specific treatment recommendations for infections |  | **√** |  | **√** |
|  | - Antibiotics are reviewed before being added to the medication formulary, if one exists |  | **√** | **√** |  |
|  | 26. Does your facility implemented practices to improve antibiotic consumption? If yes, indicate which practices are currently in place (select all that apply) |  | **√** |  | **√** |
|  | - Situation-Background-Assessment-Recommendation (SBAR) are used when patients are suspected having infection |  | **√** |  | **√** |
|  | -Antibiotic consumption information is communicated/received when patients are transferred to/from other health care facilities |  | **√** |  | **√** |
|  | - Reports summarizing antibiotic susceptibility patterns (e.g., antibiogram) are available and updated periodically |  | **√** |  | **√** |
|  | -Antibiotic reviews/time-outs are performed for antibiotic orders |  | **√** |  | **√** |
|  | -At least one infection-specific intervention to improve antibiotic consumption has been successfully implemented. If yes, indicate for which infection(s): |  | **√** |  | **√** |
| **Action:** Tracking | 27. Does your antibiotic stewardship program track antibiotic use? | **√** |  | **√** |  |
|  | 28.Does your antibiotic stewardship program monitor prospective audit and feedback interventions by tracking the types of interventions and acceptance of recommendations? |  | **√** | **√** |  |
|  | 29.Does your antibiotic stewardship program monitor preauthorization interventions by tracking which agents are being requested for which conditions? | **√** |  |  | **√** |
|  | 30. Does your stewardship program monitor adherence to facility specific treatment recommendations? |  | **√** |  | **√** |
|  | 31. Does your stewardship program monitor adherence to a documentation policy (dose, duration and indication)? |  | **√** | **√** |  |
|  | 32. Does your antibiotic stewardship program monitor the performance of antibiotic timeouts to see how often these are being done and if opportunities to improve use are being acted on during timeouts? |  | **√** |  | **√** |
|  | 33.Does your antibiotic stewardship program routinely perform medication use evaluations to assess courses of therapy for select antibiotics and/or infections to identify opportunities to improve use? | **√** |  | **√** |  |
|  | 34.Does your antibiotic stewardship program routinely perform medication use evaluations to assess courses of therapy for select antibiotics and/or infections to identify opportunities to improve use? | **√** |  | **√** |  |
|  | 35.Does your antibiotic stewardship program assess how often patients are discharged on the correct antibiotics for the recommended duration? |  | **√** |  | **√** |
|  | 36.Does pharmacist/s support antibiotic stewardship activities? | **√** |  | **√** |  |
|  | -Review’s antibiotic appropriateness based on agent selected, dosing regimen, duration of therapy and indication | **√** |  | **√** |  |
|  | -Establishes standards for clinical/laboratory monitoring for antibiotic-associated adverse drug events |  | **√** |  | **√** |
|  | -Review’s microbiology culture data to assess and guide antibiotic selection |  | **√** | **√** |  |
|  | 37.Does your facility monitor one or more measures of antibiotic consumption? If yes, indicate which of the following are being tracked (select all that apply) | **√** |  | **√** |  |
|  | -Adherence to clinical assessment documentation (including signs/symptoms, vital signs, physical exam findings) |  | **√** | **√** |  |
|  | -Adherence to include dose, frequency, duration and indication for antibiotic orders | **√** |  | **√** |  |
|  | -Adherence to facility-specific treatment recommendations for infections |  | **√** |  | **√** |
|  | -Point prevalence of antibiotic consumption (e.g., proportion of residents on antibiotic over a given time period) | **√** |  |  | **√** |
|  | 38.Does your facility monitor one or more outcomes of antibiotic consumption? |  | **√** |  | **√** |
|  | If yes, indicate which of the following are being tracked (select all that apply) |  |  |  |  |
|  | -Rates of antibiotic-resistant micro-organisms |  | **√** |  | **√** |
|  | -Rates of antibiotic-associated adverse drug events |  | **√** |  | **√** |
| **Reporting** | 39. Does your antibiotic stewardship program share facility and/or individual prescriber-specific reports on antibiotic use with prescribers? |  | **√** |  | **√** |
|  | If yes, indicate which of the following are being shared (select all that apply) |  |  |  |  |
|  | -Measures of antibiotic consumption at the facility |  | **√** |  |  |
|  | -Personalized feedback on antibiotic prescribing practices (shared only with individual clinical providers) |  | **√** |  |  |
|  | 40. Does your antibiotic stewardship program report adherence to treatment recommendations to prescribers (e.g., results from medication use evaluations, etc)? | **√** |  | **√** |  |
|  | 41. Has your facility distributed a current antibiogram to prescribers within 18-24 months? |  | **√** |  | **√** |
| **Education** | 42. Does your stewardship program provide education to prescribers and other relevant staff on optimal prescribing, adverse reactions from antibiotics, and antibiotic resistance? |  | **√** |  | **√** |
|  | 43. Does your stewardship program provide education to prescribers as part of the prospective audit and feedback process (sometimes called “handshake stewardship”)? |  | **√** |  | **√** |
|  | 44. Does your stewardship program provide educational resources/materials on antibiotic resistance and opportunity to improve antibiotic consumption? |  | **√** | **√** |  |
|  | If yes, indicate which of the following group is provided these educational resources/materials (select all that apply) |  |  |  |  |
|  | Physicians |  |  | **√** |  |
|  | Pharmacists |  |  | **√** |  |
|  | Nursing staff |  |  | **√** |  |
| **Additional Questions on Antibiotic Stewardship Challenges** | 45. Are there areas of antibiotic misuse in your facility? | **√** |  | **√** |  |
|  | -ASP can help address misuse (If stewardship program has not been established) |  |  |  |  |
|  | -More ASP efforts are needed to address misuse (If stewardship program has been established) | **√** |  | **√** |  |
|  | **46.** Are there barriers to starting the ASP? |  | **√** |  | **√** |
|  | If yes, list the top three barriers hindering initiation or improvement of the ASP | **-** | **-** | **-** | **-** |
|  | Which type and strategies of ASP do you recommend in your hospital? In which specific wards? Why? | FHCSH: Preauthorization, Prospective audit, research | | | |
